# Induction of IL-17A Precedes Development of Airway Hyperresponsiveness during Diet-Induced Obesity and Correlates with Complement Factor D

**DOI:** 10.3389/fimmu.2014.00440

**Published:** 2014-09-15

**Authors:** Joel A. Mathews, Allison P. Wurmbrand, Luiza Ribeiro, Felippe Lazar Neto, Stephanie A. Shore

**Affiliations:** ^1^Molecular and Integrative Physiological Sciences Program, Department of Environmental Health, Harvard School of Public Health, Boston, MA, USA

**Keywords:** complement factor D, fractalkine, high-fat diet, CCL20, IL-17A

## Abstract

Obesity is a risk factor for the development of asthma. Obese mice exhibit innate airway hyperresponsiveness (AHR), a characteristic feature of asthma, and IL-17A is required for development of AHR in obese mice. The purpose of this study was to examine the temporal association between the onset of AHR and changes in IL-17A during the development of obesity by high-fat feeding in mice. At weaning, C57BL/6J mice were placed either on mouse chow or on a high-fat diet (HFD) and examined 9, 12, 15, 18, or 24 weeks later. Airway responsiveness to aerosolized methacholine (assessed via the forced oscillation technique) was greater in mice fed HFD versus chow for 24 weeks but not at earlier time points. Bronchoalveolar lavage and serum IL-17A were not affected by either the type or duration of diet, but increased pulmonary *IL17a* mRNA abundance was observed in HFD versus chow fed mice after both 18 and 24 weeks. Flow cytometry also confirmed an increase in IL-17A^+^ γδ T cells and IL-17A^+^ CD4^+^ T (Th17) cells in lungs of HFD versus chow fed mice. Pulmonary expression of *Cfd* (complement factor D, adipsin), a gene whose expression can be reduced by IL-17A, decreased after both 18 and 24 weeks in HFD versus chow fed mice. Furthermore, pulmonary *Cfd* mRNA abundance correlated with elevations in pulmonary *Il17a* mRNA expression and with AHR. Serum levels of TNFα, MIP-1α, and MIP-1β, and classical markers of systemic inflammation of obesity were significantly greater in HFD than chow fed mice after 24 weeks, but not earlier. In conclusion, our data indicate that pulmonary rather than systemic IL-17A is important for obesity-related AHR and suggest that changes in pulmonary *Cfd* expression contribute to these effects of IL-17A. Further, the observation that increases in *Il17a* preceded the development of AHR by several weeks suggests that IL-17A interacts with other factors to promote AHR. The observation that the onset of the systemic inflammation of obesity coincided temporally with the development of AHR suggest that systemic inflammation may be one of these factors.

## Introduction

Obesity is an important risk factor for the development of asthma (1–5). Obesity-related asthma is more prevalent among women and is typically non-atopic in nature (6, 7). Importantly, in obese non-atopic asthmatics, airway hyperresponsiveness (AHR), a defining feature of asthma, can be attenuated by weight loss (6, 8). Obesity decreases the efficacy of asthma control medications (5, 9), making these patients difficult to treat. Understanding the mechanistic basis for obesity-related asthma may allow for the development of therapeutics that is effective in this population.

Innate AHR is a common feature of obese mice, suggesting that these mice may be useful in understanding the relationship between obesity and asthma. Mice that are genetically deficient in leptin or its receptor (*ob/ob* or *db/db* mice) and mice that are genetically deficient in carboxypeptidase E, an enzyme involved in processing neuropeptides involved in eating behaviors (*Cpe^fat^* mice) each exhibit AHR compared to age- and gender-matched wildtype (WT) mice (10–13). Mice rendered obese by placing them on high-fat diets (HFD) also develop AHR over time (14, 15).

IL-17A has been linked to the development of innate AHR in obese mice (15): compared to chow, HFD feeding results in both obesity and AHR in WT mice, whereas AHR is not observed in mice deficient in IL-17A despite equal induction of obesity. To further evaluate the role of IL-17A, we examined the temporal association between the development of AHR and changes in IL-17A in C57BL/6J mice fed chow or a HFD for up to 24 weeks. Our results indicated an increase in *Il17a* mRNA abundance in lung tissue that preceded the development of AHR in HFD versus chow fed mice.

A previous microarray analysis comparing gene expression in lung tissue from obese *Cpe^fat^* versus lean WT mice identified several genes that were significantly affected by obesity (16). Because of the requirement for IL-17A for induction of AHR by HFD (15), we searched for evidence linking IL-17A to expression of these genes to assist in determining how IL-17A might lead to AHR. Among these genes, we identified two, *Cfd* (complement factor D/adipsin) and *Cx3cl1* (fractalkine), whose expression is reported to be affected by IL-17A (17, 18). Hence, we also examined the temporal association between the development of AHR, pulmonary *Il17a* mRNA expression, and pulmonary *Cfd* and *Cx3cl1* mRNA expression in mice fed chow or HFD for up to 24 weeks. There was no effect of HFD on pulmonary *Cx3cl1* expression, but pulmonary *Cfd* expression significantly declined in HFD versus chow fed mice, consistent with previously reported declines in pulmonary *Cfd* expression in *Cpe^fat^* mice (16). Moreover, changes in *Cfd* coincided temporally with changes in *Il17a*, and our results indicate a significant correlation between pulmonary *Cfd* expression and both pulmonary *Il17a* and AHR, suggesting that changes in pulmonary *Cfd* expression contribute to the ability of IL-17A to promote obesity-related AHR.

## Materials and Methods

### Animals

This study was approved by the Harvard Medical Area Standing Committee on Animals. Male C57BL/6J mice were placed on a HFD [Research diet (D12451)] or control normal chow (PicoLab, LabDiet, St. Louis, MO) at weaning (approximately 3 weeks of age). Mice were kept on the diet for 9, 12, 15, 18 or 24 weeks. *Db/db* and WT (C57BL/6J) controls were purchased from The Jackson Laboratories and housed for 4 weeks before use, during which time they were fed a standard mouse chow diet. *Db/db* mice and their WT controls were 10 weeks old at the time of study.

### Protocol

Baseline pulmonary mechanics and airway responsiveness to inhaled aerosolized methacholine were assessed in otherwise unchallenged chow fed or HFD fed mice. Mice were assessed 9, 12, 15, 18, or 24 weeks after initiation of the diet. After lung function measurements, mice were euthanized, blood was collected by right ventricular puncture, and bronchoalveolar lavage (BAL) was performed. The lungs were then flushed of blood by injecting 10 ml of cold PBS through the right ventricle after creating a large excision in the left ventricle. The left lung was excised and used for flow cytometry. The right lung was excised and placed in RNAlater (Qiagen, Germantown, MD, USA) for subsequent preparation of RNA for real time PCR.

### Measurement of pulmonary mechanics and airway responsiveness

Mice were anesthetized with sodium pentobarbital (50 mg/kg) and xylazine (7 mg/kg) and instrumented for the measurement of pulmonary mechanics and airway responsiveness to methacholine by the forced oscillation technique using a Flexivent system (SciReq, Montreal, QC, Canada). The chest wall was opened bilaterally to expose the lungs to atmospheric pressure and a positive end expiratory pressure of 3 cm H_2_O was applied. Volume history was standardized by thrice inflating the lungs to 30 cm H_2_O airway opening pressure. Pulmonary mechanics were then assessed after inhalation of aerosolized PBS and after successive aerosolizations of methacholine in concentrations increasing in half log increments from 0.3 to 100 mg/ml. Inflation to 30 cm H_2_O followed completion of measurements at each concentration and the next aerosolization was initiated 1 min after this inflation. The following parameters were measured, as previously described (19), every 15 s for 3 min after each concentration of methacholine: Newtonian resistance (Rn), which largely reflects the conducting airways, and the coefficients of lung tissue damping (G) and lung tissue elastance (H), which reflect changes in the lung tissue, including airway closure. At each concentration of methacholine, the three highest values of Rn, G, and H were averaged and used to construct dose response curves. We also calculated the effective concentration of methacholine required to double G (EC200G) by log linear interpolation between the two doses bounding the point where G is doubled.

### Bronchoalveolar lavage

Bronchoalveolar lavage was performed by twice instilling and withdrawing 1 ml of PBS. BAL was spun and the pelleted cells counted as previously described (20). BAL supernatant was stored at -80°C until assayed for complement factor D (adipsin, Apcam, Cambridge, MA, USA), IL-17A (Biolegend, San Diego, CA, USA), and IL-23 (eBioscience, San Diego, CA, USA).

### Real time qPCR

Total RNA was prepared as previously described (21). RNA concentration and purity was determined using a small volume spectrophotometer (Nanodrop, Thermo Scientific, USA). RNA was converted into cDNA using a commercial kit (SuperScript III for qRT-PCR, Invitrogen). All expression values were normalized to 36B4 expression using the ΔΔCt method. Primers for *Rplp0* (36B4), *Cfd, Cx3cl1, Il23*a (p19), *Il17a*, and *Ccl20* (MIP3α) have all been described previously (16, 22). Primers for *Il1α* were forward – cggcaaagaaatcaagatgg and reverse ttcagagagagatggtcaatgg; for Il1β forward – ctgtgtctttcccgtggacc and reverse – cagctcatatgggtccgaca; and for IL-6 forward – ccggagaggagacttcacag and reverse – cagaattgccattgcacaac.

### Flow cytometry

Left lungs were harvested and placed on ice in RPMI 1640 media containing 2% FBS and HEPES. Lungs were digested, prepared for flow cytometry, and analyzed as previously described (22). The following antibodies were used: Alexa Fluor 647 anti-IL-17A (clone: TC11-18H10.1), PE anti-TCRδ (clone: GL3), PE-cy7 anti-CD45 (clone: 30-F11), and Alexa Flour 488 anti-CD4 (clone: GK1.5) (all antibodies from Biolegend). These antibodies were used to determine the total number of CD4 cells, γδ T cells, IL-17A^+^ γδ T cells, IL-17A^+^CD45^+^ cells, and IL-17A^+^CD4^+^ T cells.

### Serum cytokines

Serum was prepared from harvested blood using microtainer tubes (Becton Dickinson, NJ, USA) and stored at −80°C until assayed. Serum cytokines and chemokines were assayed by multiplex assay as previously described (10, 16) (Eve Technologies, Calgary, AB, Canada). We used ELISA to assay serum IL-17A and TNFα (Biolegend for IL-17A and R&D Systems for TNFα).

### Statistics

Data were analyzed by factorial ANOVA using STATISTICA software (StatSoft, Tulsa, OK, USA), with diet and weeks on diet as main effects. Fisher’s least significant difference test was used as a *post hoc* test. A *p* value <0.05 was considered statistically significant.

## Results

### Body mass

**Figure 1:**
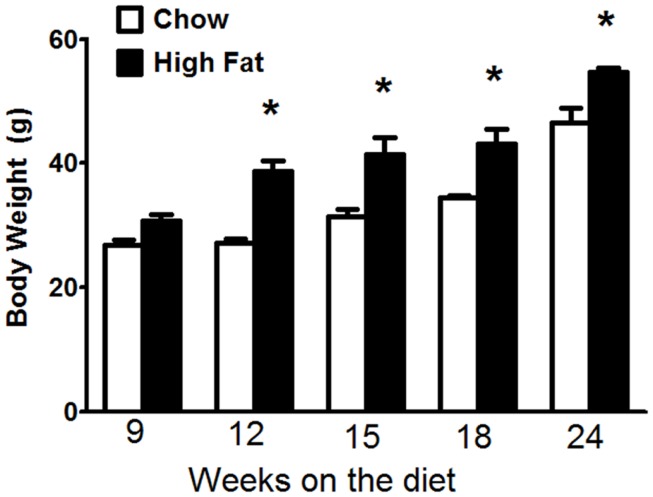
**Mice fed a high-fat diet developed obesity**. Body mass in C57BL/6J mice fed mouse chow or a high-fat diet (HFD) for up to 24 weeks. Body mass was measured on the day when the mice were euthanized. Results are mean ± SE of data from 6 to 12 mice per group. *p < 0.01 versus chow fed mice with the same duration of diet.

Factorial ANOVA indicated that both the type (*p* < 0.001) and duration (*p* < 0.001) of the diet had a significant effect on body mass (Figure 1). In both chow fed and HFD fed mice, body mass increased with time. Additionally, body mass was significantly higher in the HFD than the chow fed mice at all-time points except 9 weeks.

### Pulmonary mechanics and airway responsiveness

**Figure 2:**
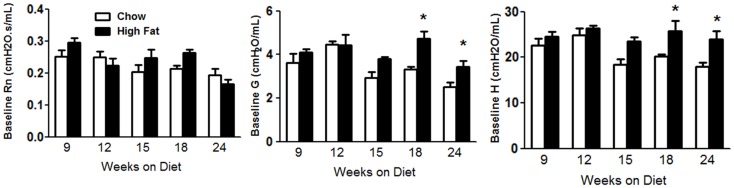
**High-fat feeding increased baseline pulmonary mechanics**. Baseline pulmonary mechanics in mice fed chow or HFD for up to 24 weeks. Rn, Newtonian resistance; G and H, coefficients of lung tissue damping and lung tissue elastance, respectively. Results are mean ± SE of data from five to nine mice per group. *p < 0.05 versus chow fed mice with the same duration of diet.

**Figure 3:**
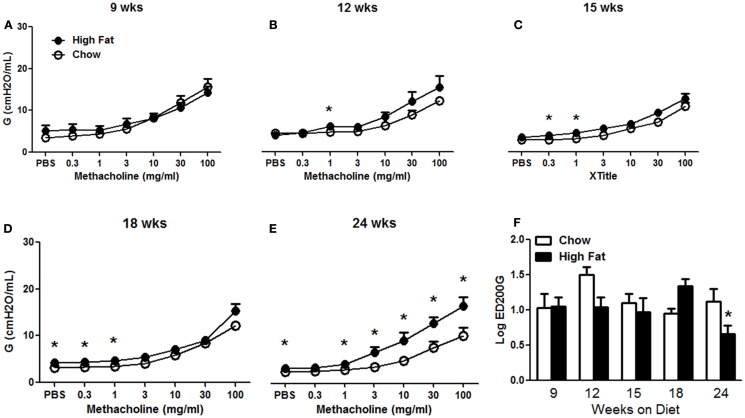
**High-fat feeding induced airway hyperresponsiveness**. **(A–E)** Airway responsiveness to inhaled aerosolized methacholine in mice fed chow or HFD for 9, 12, 15, 18, or 24 weeks. Results shown indicate changes in G, the coefficient of lung tissue damping. Similar results were obtained using H, the coefficient of lung tissue elastance. **(F)** Log concentration of methacholine required to double G (log EC200G). Results are mean ± SE of data from four to eight mice per group. *p < 0.05 versus chow fed mice with the same duration of diet.

Obesity increases baseline pulmonary mechanics in mice (10, 11, 16). We examined the development of these changes with HFD. Factorial ANOVA indicated no significant effect of HFD feeding on Rn, though there was a significant effect of time on the diet (*p* < 0.02), possibly as a result of lung and airway growth (Figure 2A). In contrast, both G and H were significantly increased in HFD versus chow fed mice (*p* < 0.01) (Figures 2B,C). Increases in G and H were observed by 18 weeks on the diet and sustained through 24 weeks. Compared to chow fed mice, HFD fed mice had significantly greater airway responsiveness and a corresponding significantly reduced EC200G after 24 weeks on the diet, whereas there was no consistent difference at earlier time points (Figure 3). Results shown in Figure 3 indicate the response to methacholine as measured by changes in G. Essentially, similar results were obtained for H, which can be impacted by airway closure, whereas no change in responsiveness was observed using Rn as the outcome indicator (data not shown).

### IL-17A is elevated in lungs of HFD versus chow fed mice

**Figure 4:**
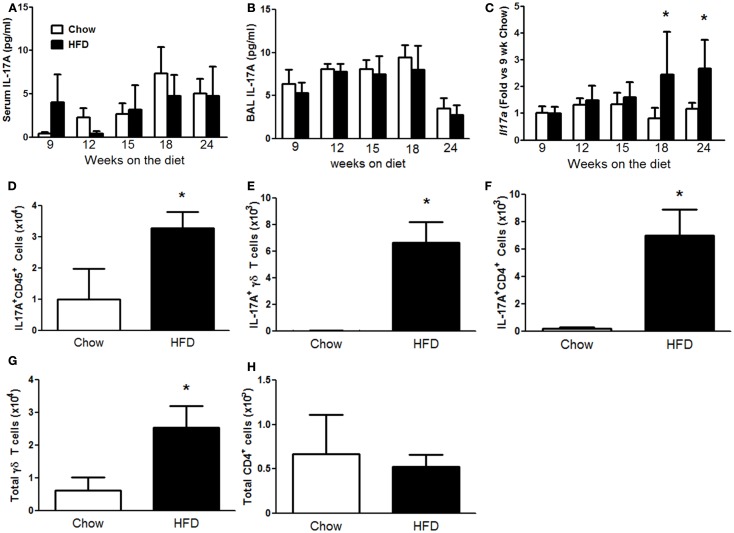
**High-fat feeding increased pulmonary IL-17A**. Serum IL-17A **(A)**, bronchoalveolar lavage (BAL) IL-17A **(B)**, and pulmonary *Il17a* mRNA expression **(C)** in mice fed chow or HFD for up to 24 weeks. **(D–F)** Total IL-17A^+^ CD45^+^ cells **(D)**, IL-17A^+^ γδ T cells **(E)**, IL-17A^+^ CD4^+^ cells **(F)**, and total γδ T cells **(G)** and CD4^+^ cells **(H)** in lungs of mice fed chow or HFD for 24 weeks. Results are mean ± SE of data from three to eight mice per group. *p < 0.05 versus chow fed mice with the same duration of diet.

Mice deficient in IL-17A do not develop AHR with high-fat feeding (15), suggesting an important role for IL-17A in the development of the innate AHR of obesity. Consequently, we explored the temporal relationship between the onset of IL-17A expression and the pulmonary phenotype induced by HFD. Neither serum nor BAL IL-17A was affected by the HFD (Figures 4A,B). In contrast, qPCR indicated greater pulmonary *Il17a* mRNA abundance in HFD versus chow fed mice after both 18 and 24 weeks on the diet (Figure 4C). Flow cytometry on cells dissociated from lungs of mice after 24 weeks on the diet confirmed an increase in IL-17A^+^CD45^+^ cells in lungs of HFD versus chow fed mice (Figure 4D) and also indicated increases in both IL-17A^+^ γδ T cells and IL-17A^+^CD4^+^ (Th17) cells (Figures 4E,F). There was also a significant increase in total γδ T cells, whereas total CD4^+^ cells were unchanged (Figures 4G,H).

**Figure 5:**
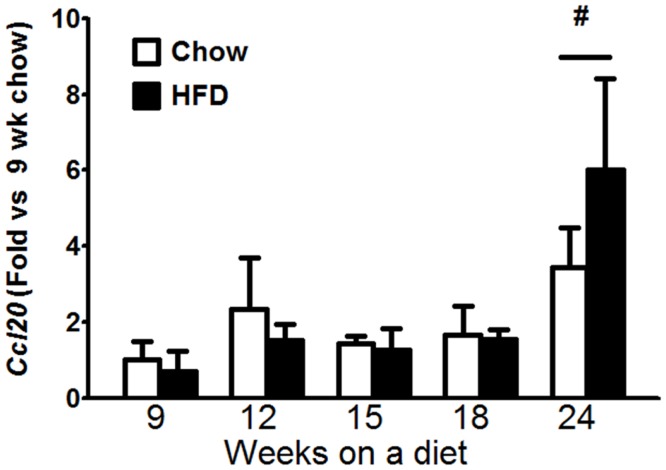
**Pulmonary *Ccl20* mRNA expression increased with time on diet**. Pulmonary *Ccl20* mRNA expression in mice fed chow or HFD for up to 24 weeks. Results are mean ± SE of data from four to eight mice per group. ^#^*p* < 0.05 versus other durations of diet

To examine potential causes for the increase in *Il17a* mRNA expression in HFD fed mice (Figure 4C), we examined the time course of changes in *Il1a, IL1b, Il23a, Il6*, and *Ccl20*. IL-1α, IL-1β, IL-6, and IL-23 are each drivers of IL-17A production (23) and CCL20 is a chemoattractant for IL-17A producing T cells (24). Factorial ANOVA indicated an effect of duration (*p* < 0.05) but not type of diet on *Ccl20* mRNA abundance. Follow up analysis indicated that the difference lay in the 24-week-mice, which exhibited increased *Ccl20* mRNA compared to other time points (Figure 5). Neither BAL IL-23 nor pulmonary *Il23a* mRNA expression was impacted by the type or duration of dietary feeding, nor was there any impact on pulmonary *Il1a, IL1b*, or *Il6* mRNA expression (data not shown).

### Possible role of adipsin

**Figure 6:**
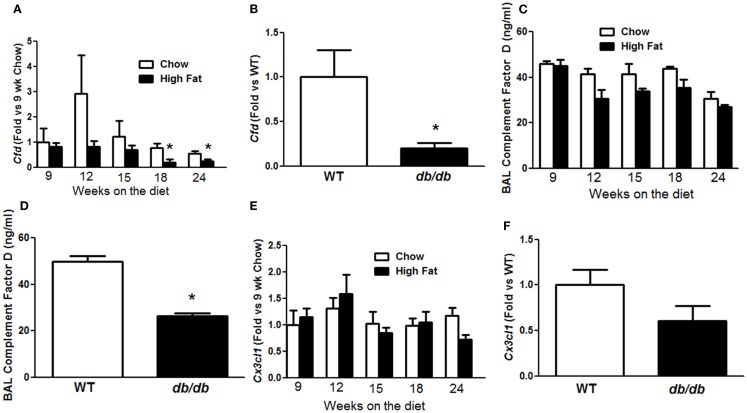
**Obesity decreased pulmonary complement factor D**. Pulmonary *Cfd* **(A,B)** and *Cx3cl1* **(E,F)** mRNA expression in mice fed chow or HFD for up to 24 weeks **(A,E)** or in lean wildtype (WT) versus obese *db/db* mice **(B,E)**. BAL complement factor D (adipsin) in mice fed chow or HFD for up to 24 weeks **(C)** or in WT versus *db/db* mice **(D)**. Results are mean ± SE of data from three to eight mice per group. *p < 0.05 versus chow fed mice with the same duration of diet or in *db/db* versus WT mice.

**Figure 7:**
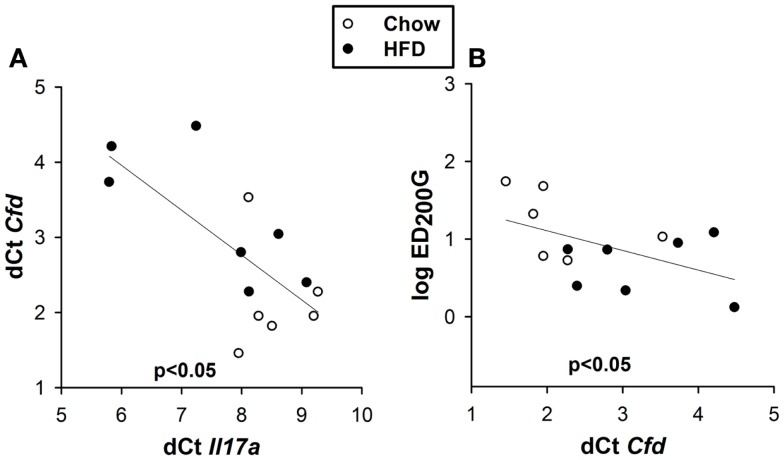
**Pulmonary *Cfd* correlates with pulmonary *Il17a* and AHR**. Correlation between pulmonary *Cfd* and *Il17a* mRNA expression **(A)** and between log EC200G and pulmonary *Cfd* mRNA expression **(B)** in mice fed chow or HFD for 24 weeks. Note that reductions in EC200G indicate increased airway responsiveness and that increases in *dCt* indicate reductions in the expression of that gene.

To determine how IL-17A might lead to AHR, we revisited a microarray analysis of genes whose expression is significantly different in lungs of obese *Cpe^fat^* versus lean WT mice (16). Among these genes, two genes, *Cfd* and *Cx3cl1*, whose mRNA expression levels are reduced in lungs of *Cpe^fat^* versus WT mice (10, 16), have also been shown to be impacted by IL-17A. In particular, IL-17A reduces *Cfd* mRNA expression in adipose tissue (18) and reduces *Cx3cl1* expression in ocular endothelial cells (21). qPCR confirmed a time-dependent decrease in pulmonary *Cfd* mRNA abundance in HFD versus chow fed mice (Figure 6A), consistent with previous observations in lungs of *Cpe^fat^* mice. Significant differences were observed at 18 and 24 weeks of diet (Figure 6A), the same time points where diet-related changes in *Il17a* mRNA were observed (Figure 4C). Because we have also observed innate AHR in *db/db* versus WT mice, we also measured *Cfd* expression in *db/db* mice to determine if changes in pulmonary *Cfd* expression were consistent across the multiple forms of obesity that exhibit AHR. We observed significantly reduced *Cfd* mRNA expression in lungs of obese *db/db* versus lean WT mice (Figure 6B). BAL adipsin was also reduced in both HFD versus chow fed mice (*p* < 0.01) and in *db/db* versus WT mice (*p* < 0.05) (Figures 6C,D). Importantly, in the 24-week-mice, there was a correlation between *Cfd* mRNA and *Il17a* mRNA expression (Figure 7A) and between *Cfd* mRNA and the EC200G (Figure 7B), an index of AHR. In contrast, there was no significant difference in pulmonary *Cx3cl1* mRNA abundance between chow fed and HFD fed mice, or between WT and *db/db* mice, although there was a trend toward reduced *Cx3cl1* mRNA abundance in both types of obesity (Figures 6E,F).

### Systemic inflammation

**Figure 8:**
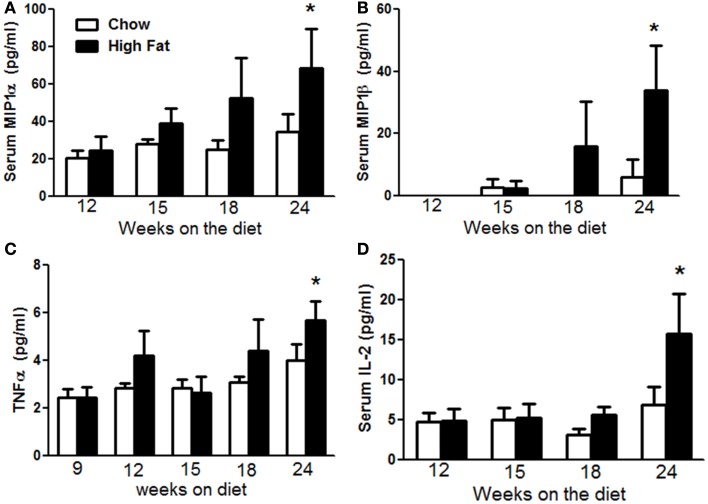
**High-fat feeding caused systemic inflammation**. Serum MIP-1α **(A)**, MIP-1β **(B)**, TNFα **(C)**, and IL-2 **(D)** in mice fed chow or HFD for up to 24 weeks. Results are mean ± SE of data from six to nine mice per group. *p < 0.05 versus chow fed mice with the same duration of diet.

Low grade systemic inflammation is now recognized as a common feature of obesity and has been shown to contribute to a wide variety of the comorbidities of obesity including type 2 diabetes (25) and atherosclerosis (26). Increases in pulmonary *Il17a* expression preceded the induction of AHR by several weeks (Figure 4C), suggesting the additional involvement of factors that developed more slowly. To determine if obesity-induced systemic inflammation might be one of these factors, we measured multiple cytokines in the serum by multiplex assay over the course of development of obesity. Factorial ANOVA indicated that compared to chow feeding, high-fat feeding caused a time-dependent increase in serum MIP-1α, MIP-1β, and TNFα (*p* < 0.05 in each case) (Figures 8A–C), consistent with observations of others (25, 27, 28). While there was a trend toward increases in all three of these moieties after 18 weeks on the diet, the effect did not achieve statistical significance until 24 weeks. We also observed significantly greater IL-2 in serum of 24-week-old HFD versus chow fed mice (Figure 8D).

## Discussion

Body mass was significantly greater in HFD than chow fed mice after 12 weeks of diet (Figure 1). This difference was maintained through 24 weeks. Nevertheless, compared to chow fed mice, HFD fed mice did not develop changes in pulmonary mechanics until 18 weeks (Figure 2) and did not develop AHR until 24 weeks on the diet (Figure 3), even though the magnitude of differences in body mass was no greater at this time than at 12 weeks of diet (Figure 1). This delay between the onset of obesity and the onset of AHR is consistent with previous observations in HFD fed mice (14), and may be the result of the time required for the induction of conditions necessary for recruitment of IL-17A^+^ cells to the lung. IL-17A is required for the development of AHR in HFD fed mice (15), and we also observed a delay between the onset of increases in body mass (Figure 1) and the onset of increases in pulmonary *Il17a* expression (Figure 4C). Indeed, the induction of significant changes in pulmonary *Il17a* expression (Figure 4C) by HFD coincided with increases in pulmonary mechanics (Figure 2) and preceded the development of AHR by several weeks (Figure 3).

Our data suggest that obesity-related reductions in pulmonary *Cfd* expression may contribute to effects of IL-17A that promote obesity-related AHR. Obesity-related AHR is observed not just in mice rendered obese by high-fat feeding (Figure 3) but also in obese *db/db* (11) and obese *Cpe^fat^* mice (29). Similarly, pulmonary expression of *Cfd* was reduced in all three types of obese mice [Figures 6A,B for HFD and *db/db* mice and (10, 16) for *Cpe^fat^* mice]. Decreased complement factor D has also been reported in the serum and adipose tissue (30) and in the liver (31) of obese mice. Importantly, changes in pulmonary *Cfd* expression coincided temporally with changes in pulmonary *Il17a* expression (Figure 4C). Additionally, we observed a significant inverse correlation between pulmonary expression of *Cfd* and *Il17a* (Figure 7A) in the 24-week-mice (at which time AHR was present in the HFD mice). The correlation between *Il17a* and *Cfd* (Figure 7A) is consistent with reports of decreased *Cfd* expression in adipocytes treated with IL-17A (18). Importantly, pulmonary *Cfd* expression also correlated with AHR (Figure 7B).

The mechanistic basis for the relationship between pulmonary *Cfd* expression and AHR is not established. Complement factor D cleaves factor B after it has bound to C3 (H20) resulting in activation of the alternatively activated complement pathway (32). Others have reported reduced allergen induced AHR in factor B-deficient and C3a-deficient mice. These data suggest that attenuated activation of the alternative pathway, as would be expected with the reduced *Cfd* expression observed in obese mice [Figures 6A,B (31, 33)], would reduce, not augment airway responsiveness as observed in obese mice (Figure 3). However, there is some evidence that complement can also serve a protective role in the lung. Lung injury occurs in multiple models of liver injury and depletion of complement with cobra venom factor in mice with liver injury increases NF-κB activation and inflammation in the lung (34), events that might be expected to promote AHR. In this context, it is important to note that liver pathology is a common feature of obese mice (35). In addition, using a bioinformatics approach, Couto Alves et al. (36) identified significant interactions between T cell activation and the complement system in patients with allergic rhinitis, including reductions in expression of most complement species, including CFD.

Increases in serum IL-17A are observed both in *Cpe^fat^* mice (16) and in *db/db* mice (unpublished observations) and serum IL-17A is also elevated in obese human subjects (37). However, there was no change in serum IL-17A with either the type or duration of diet (Figures 4A,B). The substantially greater increases in body mass extant in the genetically obese *Cpe^fat^* and *db/db* mice than in HFD mice may explain this apparent discrepancy. However, the absence of increases in serum IL-17A (Figure 4A) despite increases in AHR (Figure 3) with HFD, in conjunction with the observations that obesity-related AHR does not occur in IL-17A-deficient mice (15), suggest that the source of the IL-17A that is important for AHR is the lung not the blood. Indeed, we observed increases in IL-17A^+^ cells in the lung tissue of HFD versus chow fed mice (Figure 4). IL-17A expressing cells were only examined at 24 weeks, the time point at which we observed AHR (Figure 3). We did not examine ILC17 cells, but did observe increases in both IL-17A^+^ γδ T cells and Th17 cells (Figure 4), consistent with the observations of others (15). The observation that IL-17A producing cells are increased by HFD feeding is not unique to the lung. Compared to chow, HFD also increases the number of IL-17A^+^ cells in other organs and tissues, including spleen, liver, and joints (38–40).

We found no evidence of a role for either IL-6 or IL-23 in the induction of pulmonary IL-17A^+^ T cells during HFD. Instead, leptin, an adipose-derived hormone that increases in obesity may be involved. The mechanistic basis for effects of IL-6 and IL-23 on IL-17A involves activation of STAT3 (41), and leptin also induces STAT3 activation (42). Indeed, leptin receptors are expressed on T cells and leptin can induce the differentiation of T cells into IL-17A producing cells (43). We also observed increased pulmonary mRNA expression of *Ccl20*, a chemoattractant for IL-17A producing cells, after 24 weeks (Figure 5), though there was no significant difference in *Ccl20* expression between chow and HFD mice. Such increases, in conjunction with leptin-mediated increases in circulating IL-17A^+^ T cells in the HFD mice, would be expected to increase the number of IL-17A^+^ cells in the lungs.

The co-incident changes in pulmonary mechanics (Figure 2) and pulmonary *Il17a* expression (Figure 4C) may be the result of direct effects of IL-17A on airway smooth muscle that promote contractility (44). Increases in G and H occur not only with HFD but also in *Cpe^fat^, db/db*, and *ob/ob* mice (10, 11, 13, 16), and may be the result of small airway closure, a phenomenon that also occurs in human obesity (45–48). However, the observation that increases in pulmonary *Il17a* expression preceded the development of AHR suggests that factors in addition to IL-17A are required for the induction of AHR. Our data suggest that the systemic inflammation of obesity may be one of these factors. There was a delay between the onset of increases in body mass and the onset of systemic inflammation (Figure 8) consistent with previous reports (49), but the onset of systemic inflammation (Figure 8) was co-incident with the development of AHR (Figure 3), both being observed at 24 weeks of diet but not earlier. In other systems, IL-17A synergizes with other cytokines including TNFα to promote changes in cell function (50) and similar synergistic effects of TNFα and IL-17A may be necessary to drive AHR in obesity. The observations that both IL-17A (15) and TNFR2 (16) are required for the development of AHR in obese mice are consistent with this hypothesis.

In conclusion, it has been previously established that IL-17A is required for obesity-related AHR. Data presented here extend those observations by showing that pulmonary rather than systemic IL-17A is important for obesity-related AHR and suggest that changes in pulmonary *Cfd* expression contribute to the AHR-promoting effects of IL-17A. Further, the observations that increases in pulmonary *Il17a* mRNA expression preceded, whereas the onset of the systemic inflammation of obesity coincided temporally with the development of AHR suggest that systemic inflammation may interact with IL-17A to promote AHR.

## Conflict of Interest Statement

The authors declare that the research was conducted in the absence of any commercial or financial relationships that could be construed as a potential conflict of interest.
